# Cellular Stress Following Water Deprivation in the Model Legume *Lotus japonicus*

**DOI:** 10.3390/cells1041089

**Published:** 2012-11-13

**Authors:** Marco Betti, Carmen Pérez-Delgado, Margarita García-Calderón, Pedro Díaz, Jorge Monza, Antonio J. Márquez

**Affiliations:** 1 Department of Vegetal Biochemistry and Molecular Biology, Chemistry Faculty, University of Seville, Apartado 1203, 41071-Sevilla, Spain; Email: cmperez@us.es (C.P.-D.); marbioq@us.es (M.G.-C.); cabeza@us.es (A.J.M.); 2 Biochemistry Laboratory, Department of Vegetal Biology, Agronomy Faculty, Av. E. Garzón 780, CP 12900 Montevideo, Uruguay; Email: pediaz@fagro.edu.uy (P.D.); monzajorge@gmail.com (J.M.)

**Keywords:** drought stress, *Lotus japonicus*, transcriptomics, cellular stress response, reactive oxygen species, transcription factors

## Abstract

Drought stress is one of the most important factors in the limitation of plant productivity worldwide. In order to cope with water deprivation, plants have adopted several strategies that produce major changes in gene expression. In this paper, the response to drought stress in the model legume *Lotus japonicus* was studied using a transcriptomic approach. Drought induced an extensive reprogramming of the transcriptome as related to various aspects of cellular metabolism, including genes involved in photosynthesis, amino acid metabolism and cell wall metabolism, among others. A particular focus was made on the genes involved in the cellular stress response. Key genes involved in the control of the cell cycle, antioxidant defense and stress signaling, were modulated as a consequence of water deprivation. Genes belonging to different families of transcription factors were also highly responsive to stress. Several of them were homologies to known stress-responsive genes from the model plant *Arabidopsis thaliana*, while some novel transcription factors were peculiar to the *L. japonicus* drought stress response.

## 1. Introduction

The study of the plant response to drought stress is very important. The alarming growth rate of the world’s population, which depends mostly on plants for food energy intake, has led to an increased demand for crops with improved productivity. Drought stress, together with salinity, is one of the most important factors that reduces the yield of crops world-wide. Alongside classical breeding programs, functional genomics approaches are fundamental for the generation of plants with increased resistance to drought. A first step for the isolation of genes that may be related to drought tolerance is the identification of drought-responsive genes in the plant species considered. This can be now carried out thanks to the availability of DNA microarrays for several plant species. In this way, a great number of drought responsive genes have been identified, especially in the model plant *Arabidopsis thaliana* [1], and their contribution to drought or stress tolerance can be assessed in the laboratory. Genes that improve abiotic stress tolerance mostly encode for transcription factors, enzymes for the biosynthesis of sugars and compatible solutes, proteins of the antioxidant defense and ion transporters, among others. 

Exposure to water shortage, especially when followed by rapid dehydration, triggers the induction of basic responses aimed to reduce water loss and the concomitant oxidative stress associated with it [2]. However, many more genes are induced during drought, as demonstrated by several transcriptomic studies. This includes the genes involved in stress sensing and signal transduction, together with several metabolic pathways that are modulated in order to maximize the fitness of the plant under water deprivation [1,3]. The vast number of genes that are modulated by water deprivation illustrates the severe stress conditions caused by drought at the cellular level. At the level of whole plant metabolism, severe drought causes inhibition of photosynthesis and a general metabolic dysfunction that compromises plant growth and fertility, and can lead to premature senescence [4]. Cellular responses to drought include the adjustment of the membrane system, which may be compromised under stress, as well as important changes in the cell cycle and cell division [4]. Several compounds and macromolecules are produced in order to deal with the water loss and the excess of reactive oxygen species produced. This includes chaperonins like heat-shock proteins and compatible solutes, and small molecules such as proline, glycine betaine and raffinose, which play several protective roles, for example, in helping to maintain cell turgor and scavenging reactive oxygen species (ROS) [1]. Other stress-responsive proteins produced under water deprivation include the late embryogenesis-abundant (LEA) proteins, which have a protective role during dehydration, aquaporins; these form pores in the lipid bilayer and facilitate water flux and proteases, which are produced in order to get rid of damaged proteins and to remobilize nitrogen [1,5]. 

Among different plant species, the Leguminosae are second only to the Gramineae in importance to humans as a source of food, feed for livestock and as raw materials for industry [6]. The productivity of legumes can be hampered by drought stress, since this condition strongly limits nitrogen fixation in the nodules [7]. Unfortunately, cultivated legume species are poor models for genomic research. In fact, some of them are tetraploids and many have large genome sizes and are recalcitrant to transformation [7]. As a consequence, two legumes species, *Lotus japonicus* and *Medicago truncatula*, have been adopted internationally as models for legume research. In particular, *L. japonicus* serves as a model for the study of several other species of the genus Lotus that are highly used as pasture in temperate regions [8], where the plants can be exposed to sudden periods of drought. The response of *L. japonicus* to different kinds of abiotic stress has been studied at the transcriptomic, metabolomic and proteomic levels [9,10,11,12]. Several of these studies have been carried out thanks to the recent availability of an Affymetrix Genechip designed specifically for *L. japonicus*. 

Previous work from our group demonstrated the important role played by the plastidic isoform of glutamine synthetase (GS) of *L. japonicus* in the response to drought stress and in drought-induced proline production [10,13]. These results were obtained by comparing the drought-stress transcriptomes of wild-type (WT) and mutant plants that lacked of plastidic GS. Since plastidic GS is fundamental for the reassimilation of the ammonium generated during photorespiration, mutants that lack of this enzyme show an air-sensitivity phenotype typical of plants that are impaired in one of the steps of the photorespiratory cycle [14,15]: plants can grow well under a CO_2_-enriched atmosphere, where photorespiration is suppressed, but show several stress symptoms like chlorosis and necrosis when grown under normal air conditions. For this reason, previous transcriptomic studies that compared the response to drought of WT and plastidic GS mutants were carried out under CO_2_-enriched atmosphere. In the present work we have studied the response of *L. japonicus* plants to drought stress under physiological conditions (normal air). The transcriptomes of well-watered and drought-stressed plants grown under normal air conditions were compared and, according to the aim of this special issue, a particular attention has been paid to the cellular mechanism of response to the stress conditions imposed by water deprivation. 

## 2. Results and Discussion

### 2.1. Drought Stress Transcriptomics of the Model Legume Lotus japonicus

**Figure 1:**
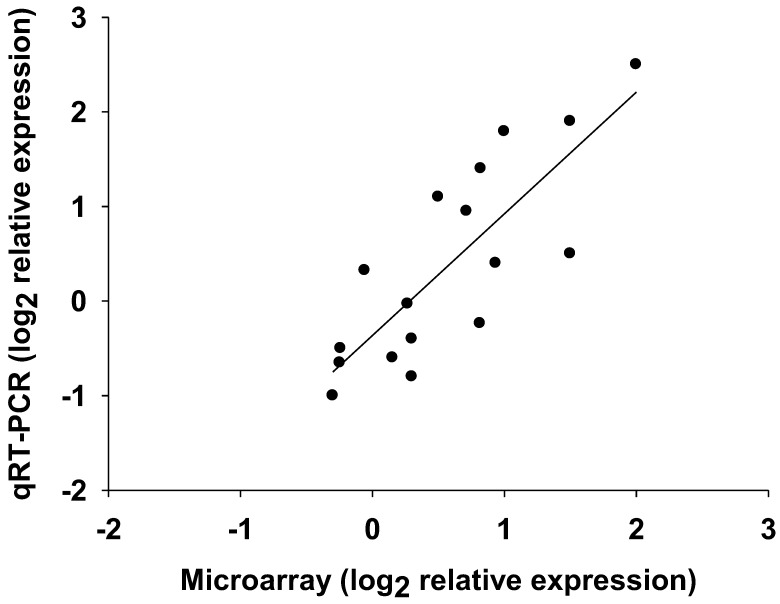
qRT-PCR validation of the microarray data. Each point represents one of the genes for proline metabolism that were previously used for the validation of Lotus microarray data [10]. The values reported in the graph are the log_2 _of the difference in expression levels between normally watered and drought-stressed plants. Linear regression analysis gave a regression coefficient of r^2^ = 0.67. Values are the mean of three independent biological replicates.

In order to study the cellular response of *L. japonicus* plants to drought stress, a water deprivation experiment was carried out with 35 days-old plants. Drought was imposed by withholding watering for 4 days. After this period the plants showed a relative water content of about 60%. As demonstrated previously, this level of water loss does not compromise the performance of WT *L. japonicus* plants, which are still able to rapidly restore their water status if watered again [10]. Longer periods of water deprivation caused death of the youngest leaves and were not considered for this study. Leaves were harvested from drought-stressed plants and normally-watered plants, used as a control. The RNAs obtained were hybridized to the Lotus1a520343 Affymetrix Genechip^®^, which contains 52,749 unique probesets. A probeset is an oligonucleotide designed to measure the expression of a known or predicted sequence of mRNA. Several probesets may correspond to the same gene, in such a way that most of *L. japonicus* gene transcripts are analyzed in a single DNA chip. Drought-induced changes in the transcriptome were analyzed by a significance-based comparison of control and drought-stressed plants, applying a false discovery rate (FDR) of less than 0.05 and using three independent biological replicates for both control and drought-stressed plants. A validation of the microarray data was carried out by qRT-PCR. The expression levels of different genes for proline metabolism that are normally highly modulated by drought [10] were determined. A good agreement between qRT-PCR and microarray data was obtained (Figure 1). 

### 2.2. Global Overview of the Dataset

In total, 3,950 genes were modulated after four days of water deprivation. The ratio between the number of induced and repressed genes was slightly biased towards induction, with 2,064 up-regulated and 1,886 down-regulated ones. The full list of the 3,950 genes that were significantly modulated by drought can be found online as supplemental material (Supplemental Table S1).

**Figure 2:**
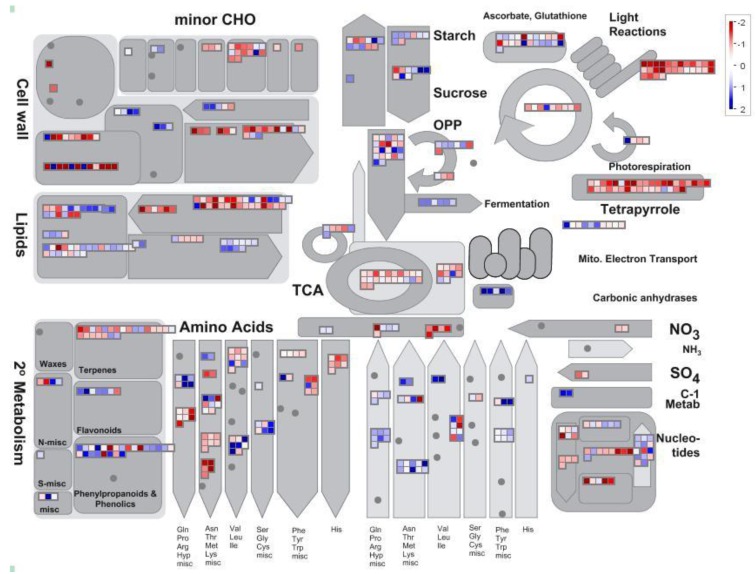
MapMan general metabolism overview of the genes modulated by four days of drought in *L. japonicus* plants. Each square corresponds to a gene. Red and blue indicate lower and higher expression than the control, respectively. The scale bar is shown in log_2_.

An overview of the different genes modulated by drought in relation to their correspondent metabolic pathways was generated using the MapMan program [16] (Figure 2). Many genes related to photosynthesis like the ones coding for the structural component of the photosystems (“Light Reactions” in Figure 2) and for the biosynthesis of photosynthetic pigments (tetrapyrroles) were repressed as a consequence of water deprivation, indicating that there is a general shutdown of photosynthetic metabolism in *L. japonicus* in response to drought. The central carbon metabolism was also affected by drought conditions, with a general repression of the genes encoding for the enzymes of the TCA cycle. Modulation of both lipid biosynthesis and degradation was suggestive of reorganization of membrane composition and/or of membrane damage. 

Consistent with these results was the increased level of lipid peroxidation previously observed in *L. japonicus* under drought conditions [10]. Several pathways for the biosynthesis and degradation of amino acids were also modulated by drought stress. Among them, genes encoding for pyrroline-5-carboxylate synthetase (P5CS) were induced (Supplemental Table S1). P5CS catalyzes the first step in the biosynthesis of proline, an amino acid that is normally accumulated in plant cells in response to different kinds of abiotic stresses [17]. The induction of other genes involved in the production of compatible solutes like trehalose and γ-aminobutyrric acid suggested an increased production of these molecules. A gene encoding for trehalose-6-phosphate synthase was induced about two-folds (probeset Ljwgs_070708.1; Supplemental Table S1). Interestingly, overexpression of this gene in tobacco plants lead to increased drought tolerance [18]. The data obtained indicate that *L. japonicus* plants undergo an extensive reprogramming of the transcriptome in response to drought stress. Considering the great number of genes and pathways affected by water deprivation, a further analysis of the dataset was carried out focusing on the identification of the genes and metabolic pathways that were most significantly modulated. 

### 2.3. Analysis of the Most Modulated Genes

Highly stress-responsive genes are good candidates for the evaluation of their contribution to drought tolerance in targeted studies, either by overexpression of the candidate gene or by the obtention of specific mutants [1]. In the case of *L. japonicus*, the development of a TILLING reverse genetic tool [19] and, much more recently, of a population of insertion mutants created using the LORE1 endogenous retrotransposon [20] allow the rapid obtention of mutants in a selected gene. For this reason, we focused our analysis on the top 10 up- and down-regulated genes in the drought-stress transcriptome (Table 1). The gene sequences were blasted against the current databases and the TAIR database [21] and the corresponding Arabidopsis orthologous genes were identified. 

The most drought-induced gene is ortholog to the Arabidopsis 16 kDa outer plastid envelope protein Oep16. This gene was also among the most induced by drought under CO_2_-enriched atmosphere [13]. The corresponding protein product belongs to a family of pre-protein and amino acid transporters present in chloroplasts and mitochondria of plants, as well as in bacteria [22]. Proteins of the mitochondrial and plastidic protein import machineries are often modulated by different kinds of abiotic stresses [23]. Considering the limited protein encoding capacity of these organelles, it is easy to understand that many of the protein and enzymes required in response to stress depend on the import of cytosol-synthesized proteins. This may explain the high induction observed for the *L. japonicus* Oep16 ortholog. The second and third most induced genes belong to the LEA family (probesets Ljwgs_133863.1 and chr1.TM0221.11). LEA genes encode for a broad group of stress-protection proteins that are expressed during embryo maturation in several plants [1], whose precise biochemical way of action is still not fully understood [5]. 

Genes involved in the antioxidant response like a glutathione-S-transferase (probeset Ljwgs_074013.2) and a nucleoredoxin (probeset Ljwgs_026189.1) were also highly induced by drought, suggesting increased oxidative stress. Nucleoredoxin are multi-domain thioredoxins, whose function remains still rather unexplored in plants [24], while glutathione-S-transferases are involved in xenobiotics detoxification, ROS scavenging and may remove peroxidized lipids [25]. A gene encoding for beta glucosidase, a protein that hydrolyzes glycosides of abscisic acid (ABA) to liberate active ABA, was highly induced (probeset chr2.CM0056.64). ABA is important in the response to drought stress since it causes stomatal closure, which prevents excessive water loss and induces the expression of stress-related genes [26]. It is worth noting that overexpression of beta glucosidase in *Arabidopsis* resulted in increased drought and salt tolerance [27]. Different genes encoding for transcription factors (TFs) were also highly modulated (Table 1). Interestingly, while a NAM, ATAF1/2 and CUC2 (NAC) domain TF ortholog to Arabidopsis NAC47 was highly induced (probeset chr1.CM0104.32), RAD-like 5 (AtRL5), a gene related to the myeloblastosis (MYB) family of TFs was the most repressed under drought stress (probeset chr2.CM0249.113). RAD-like transcription factors are a subfamily of the MYB factors. Members of the RAD-like family of TFs are involved in floral development in Arabidopsis [28]. However, the exact role of AtRL5 is unknown. On the other hand, NAC47 was described in Arabidopsis as a gene responsive to ammonium supply in a previous transcriptomic study [29]. 

**Table 1:** Top 10 genes up- or down-regulated by drought in leaves of *L. japonicus*. The fold-change (FC) is expressed as the log_2_ of the difference in relative expression levels between drought stress conditions and normal watering. The description and locus identifier of the Arabidopsis orthologous genes are also reported.

Probeset	log_2_ FC	Arabidopsis ortholog	Locus
Up-Regulated			
chr4.CM0429.5	4.57	Outer plastid envelope protein Oep16	At4g16160
Ljwgs_133863.1	4.53	LEA7	At1g52690
chr1.TM0221.11	4.24	LEA4-5	At5g06760
Ljwgs_062789.1	4.16	oxidoreductase	At5g09300
chr2.CM0056.64	4.01	Beta-glucosidase	At1g02850
Ljwgs_013141.2	3.94	Putative protein	At2g25625
Ljwgs_053770.1	3.82	Putative protein	At5g66780
Ljwgs_074013.2	3.71	Glutathione-S-transferase	At2g29490
chr1.CM0104.32	3.70	NAC47	At3g04070
Ljwgs_026189.1	3.66	Putative nucleoredoxin	At1g60420
Down-regulated			
chr2.CM0249.113	−5.14	AtRL-5	At1g19510
Ljwgs_015206.1	−4.70	Expansin	At1g26770
BM0976.11	−4.46	Retrotransposon	n.d.
Ljwgs_065092.1	−4.44	Aspartyl protease family protein	At1g03220
Ljwgs_028040.1	−4.42	AMT1;4	At4g28700
chr3.TM0745.32	−4.34	Delta tonoplast integral protein AtTIP2;1	At3g16240
chr6.CM0139.42	−4.18	Aspartyl protease family protein	At1g03220
chr3.CM0112.48	−4.16	DNAJ-like chaperone	At4g36040
Ljwgs_098953.1	−4.14	Retrotransposon	At4g27210
chr1.CM0233.42	−3.94	Transposable element	At1g35920

Among the most drought-repressed genes there was one encoding for expansin, an enzyme involved in cell-wall loosening during the enlargement of plant cells. This may indicate that the cells are undergoing cell wall restructuration under water deprivation. Other highly repressed genes encoded for proteins of the aspartyl protease family (probesets Ljwgs_065092.1 and chr6.CM0139.42), a chaperon protein (probeset chr3.CM0112.48) and two transposable elements (probesets BM0976.11 and Ljwgs_098953.1). A novel NH_4_^+^ transporter of the LjAMT1 family (probeset Ljwgs_028040.1), with 89% similarity to LeAMT1.3 from tomato was repressed more than 20-fold (2^4.42^). This transporter was also highly repressed by salt stress [9] and by drought under CO_2_-enriched atmosphere [13]. Another repressed gene related to ammonium transport (probeset chr3.TM0745.32) was homolog to the Arabidopsis delta tonoplast integral protein AtTIP2;1. This protein is involved in ammonium transport into the vacuole and the corresponding gene is induced by ammonium [30]. Further studies should be needed in order to understand why several genes related to ammonium transport are modulated in *L. japonicus* under abiotic stress conditions.

In summary, these results indicate that the most regulated genes in *L. japonicus* cells under water deprivation are involved in several aspects of cellular metabolism, including the production of protective molecules, oxidative stress response, transport, cell wall restructuration, transcription and hormone metabolism among others. Some of these processes are related to general cellular stress responses such as the deformation and damaging of membranes, lipids, proteins and DNA together with the generation of oxidative stress [31]. On the other hand, processes like cell wall restructuration and transport of water and ammonium are probably more specific to the response to drought stress. 

### 2.4. Analysis of Overrepresented Pathways

**Figure 3:**
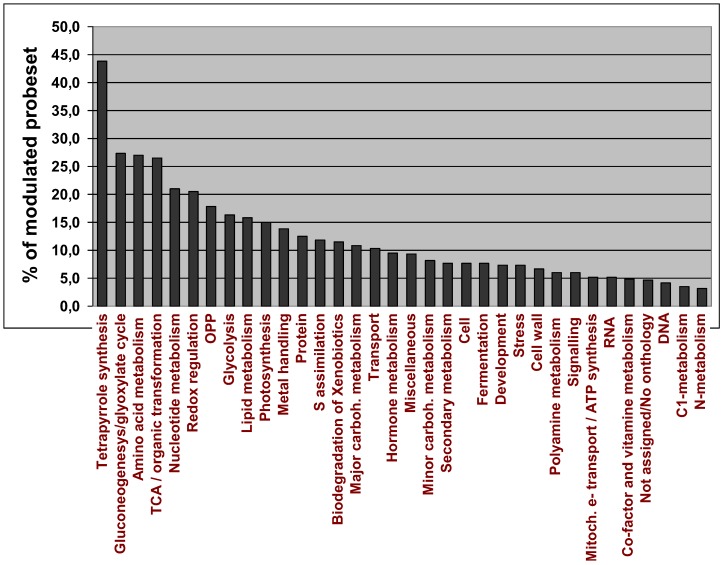
Percentage of transcripts from the 36 functional groups (or BINs) defined by the MapMan software that were significantly modulated by drought. The functional category “unassigned” was not considered in this analysis.

The effect of drought on the expression of different functional groups of genes was tested. The percentage of the total number of genes modulated by drought within each functional category is indicated in Figure 3. 16 of the 36 functional groups defined by the MapMan software showed a modulation of at least 10% of their total genes, confirming that drought induces an extensive reprogramming of the transcriptome. Six functional categories showed changes in the expression of more than 20% of their members: tetrapyrrole synthesis (where almost the 45% of the genes were modulated), gluconeogenesis/glyoxylate cycle, amino acid metabolism, TCA cycle, nucleotide metabolism and redox regulation. 

**Figure 4:**
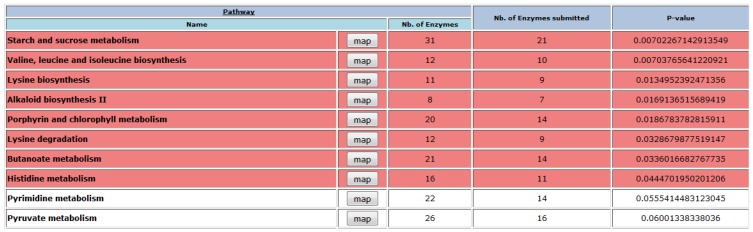
Analysis of the dataset using Pathexpress. The significantly (*p* < 0.05) over-represented pathways are highlighted in red.

In order to determine if the high modulation of these metabolic pathways was statistically significant, the dataset was analyzed using the program Pathexpress [32]. This algorithm allows the identification of the most relevant metabolic pathways within a group of genes. Using a P cutoff value of less than 0.05, the program identified eight over-represented pathways (Figure 4). First of all, the analysis carried out with Pathexpress confirmed that the biosynthesis of photosynthetic pigments was highly repressed under drought conditions. This down-regulation of photosynthetic metabolism observed is a common response to high levels of stress [33] and may suggest a decrease in photosynthesis in *L. japonicus* under drought. The other over-represented metabolic routes felt mainly under the categories of carbon and amino acids metabolism, in good agreement with the data presented in Figure 3. Of particular interest was the fact that the pathways for both lysine biosynthesis and degradation were highly regulated. Several genes for lysine biosynthesis were repressed, while genes for lysine degradation were induced, suggesting a decrease in the lysine pool as a consequence of drought. The repression of the biosynthetic genes of the amino acids of the aspartate family (that includes lysine) and the concomitant induction of the corresponding catabolic genes is a general regulatory strategy observed in plant abiotic stresses that cause energy deprivation [34]. Under such conditions, lysine degradation may contribute to cellular energy metabolism by providing carbon skeletons to fuel the TCA cycle [35]. 

Taken together, these results indicate that the metabolic pathways that are more regulated by drought stress in *L. japonicus* are related to carbon and amino acid metabolisms. Drought stress, like other kind of abiotic stresses, induces stomatal closure, which reduces the photosynthetic rate and affects the rate of CO_2_ assimilation and energy production [2]. This, consequently, results in the over-reduction of components within the photosynthetic electron transport chain that leads to the production of ROS. The reduced expression of genes for the biosynthesis of photosynthetic pigments may then be aimed to the reduction of ROS production through a reduced activity of the components of the photosystems. On the other hand, reduced photosynthesis levels should lead to lower energy and reduced carbon availability. The modulation of starch and sucrose pathways (Figure 4) may suggest remobilization of stored carbon reserves. This, together with the degradation of amino acids, may serve to fuel the TCA cycle under drought conditions.

### 2.5. Overview of the Cellular Response to Drought Stress in Lotus japonicus

The modulation of genes involved in central metabolism and in the production of defensive molecules previously described was not the only component of the cellular response to drought. In fact, a great number of genes are involved in the perception of the stress and in the consequent transmission of the stimuli to the nucleus [26]. This is usually initiated by the activation of signaling cascades that comprehend protein kinases, calcium, phospholipids, hormones and transcription factors [1]. One of the signals that triggers these signaling cascades under different stress conditions is the production of ROS. Plant cells have developed a number of strategies in order to cope with these toxic molecules [25]. In this section, we will analyze the cellular response of *L. japonicus* to drought with a special focus on the genes encoding for antioxidant enzymes and transcription factors.

**Figure 5:**
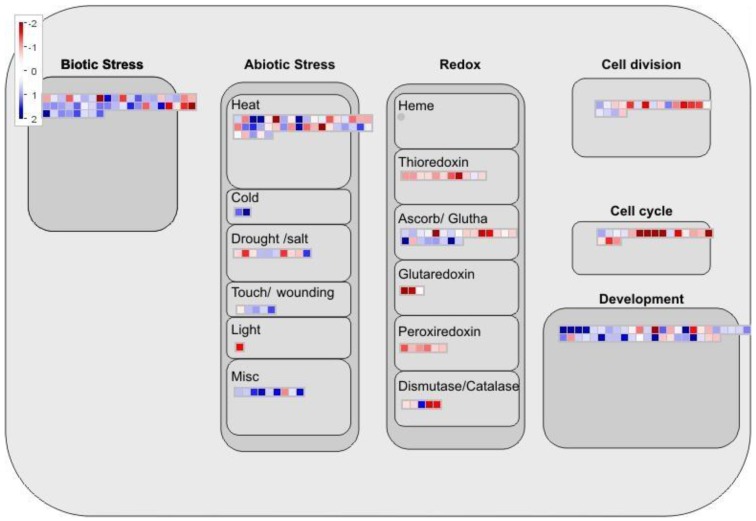
MapMan overview of the cellular response of *L. japonicus* to drought stress. Other details as described above.

Surprisingly, the cellular response to water deprivation included the modulation of several genes involved in the perception and response to other kinds of stresses in addition to drought (Figure 5). Several genes classified as responsive to biotic stress, heat, cold and wounding were recognized by the MapMan software among the modulated ones. This may be explained by the fact that the transcriptomic responses to different kinds of abiotic stresses partially overlap [36]. In addition, both biotic and abiotic stresses are also known to regulate overlapping groups of genes [37]. This is probably due to the fact that ROS, which are generated under biotic and abiotic stress, are a common signal that triggers downstream stress responses [37]. Consistent with this hypothesis is the fact that several known and unknown genes of the *L. japonicus* redox defense were regulated under drought conditions (Figure 5). Previously described redox genes that were modulated by drought included several genes coding for isoforms of glutathione peroxidase like *LjGPX1*; *LjGPX2* and *LjGPX3* (probesets chr4.CM0558.29.1, chr4.CM0558.28 and Ljwgs_038927.1 respectively) [38]. Interestingly, the expression of these three isoforms of glutathione peroxidase was not induced by salinity and was repressed by toxic metals like Cd in *L. japonicus* [38]. Other known redox genes modulated by drought were the plastidic iron superoxide dismutase (LjFeSOD1, probeset gi46402889) [39] and different isoforms of thioredoxin and peroxiredoxin [40].

The expression of several genes involved in the control of cell cycle, cell division and plant development was also altered under drought conditions (Figure 5). Several cyclins, as well as mitotic control proteins and proteins involved in cell division were present among these two groups. This is compatible with an arrest in plant growth and a decrease in the rate of cell division, both common responses of plants to drought or salinity [1].

**Figure 6:**
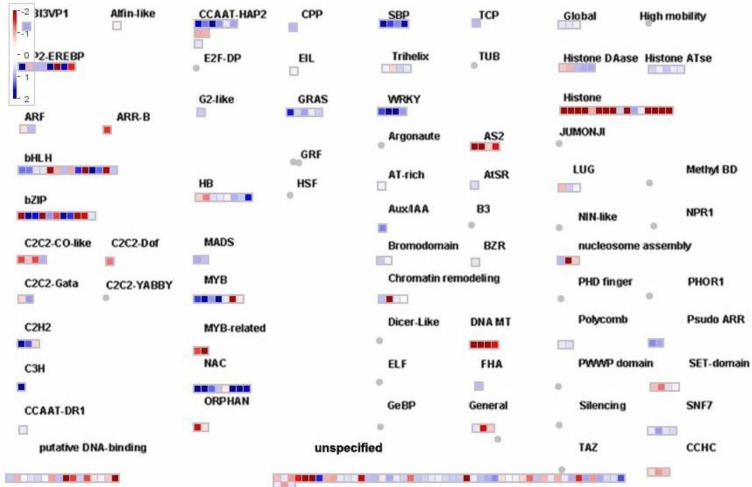
MapMan overview of the transcription factors genes that were modulated under drought conditions. Other details as described above.

An overview of the transcription factors (TFs) that responded to drought stress is presented in Figure 6. Members of several TF families that play a pivotal role in the response to drought were highly regulated, including basic leucine zipper (bZIP) domain TFs, zinc finger proteins like the basic helix-loop-helix (bHLH) family, MYB and MYB-related proteins and NAC domain TFs. Moreover, water deprivation triggered the coordinate repression of genes involved in the regulation of DNA structure and functionality like several genes encoding for histone proteins and DNA methyltransferases (DNA MT, Figure 6). This may indicate a reduced cellular division in *L. japonicus* under drought stress, as also suggested by the modulation of genes encoding for cyclins previously described (Figure 5).

The genes encoding for the most modulated TFs in response to drought stress were further analyzed (Table 2). Since legume TFs and particularly *L. japonicus* are still poorly characterized [6], the Arabidopsis orthologs to the most modulated TFs were identified. About 250 TF were identified in *L. japonicus* by the MapMan program (Figure 6). For this reason, a four-fold change threshold was applied (log_2_ of fold change > 2 or < −2) in order to consider only the most modulated genes. The identification of highly drought-responsive transcription factors is of particular interest since they may represent good candidates for the engineering of plants for improved stress resistance. Moreover, a further analysis of this group of genes was carried out by comparing the data presented here with a previously reported transcriptomic study carried out with the plastidic GS mutant *Ljgln2-2* under drought conditions [10]. Since plastidic GS is involved in the stress responsive machinery of *L. japonicus* [10], it was interesting also to determine if the same or different transcription factors were involved in the response to drought in the mutant background. 

The TF family that showed more highly induced genes was NAC, with four members induced more than four-fold in the WT (Table 2). The Arabidopsis orthologs to these NAC TFs were the previously described NAC 47 (Table 1), the NAC domain protein responsive to desiccation 26 (RD26), NAC100 and NAC1. Among these genes, only RD26 has been described as responsive to abiotic stress in Arabidopsis [41]. It is possible that some of these NAC genes are involved in the response to drought stress specifically in *L. japonicus*. Two highly induced genes were related to abscisic acid (ABA), an hormone that plays a central role in the response to drought and salinity [26]: MYB96, that regulates drought stress response by integrating ABA and auxin signals in *Arabidopsis* [42], and Aba repressor 1 ABR1 [43], a repressor of ABA-regulated gene expression. Both TFs are important for stress tolerance in Arabidopsis since overexpression of MYB96 resulted in enhanced drought resistance [42] and ABR1 mutants were hypersensitive to drought as well as other kinds of abiotic stresses [43]. These data suggest that the ABA signaling pathway under drought conditions is at least in part conserved between Lotus and Arabidopsis. Other induced genes with known Arabidopsis orthologs included the ethylene responsive transcription factor RAP2.10, AtOZF1 (oxidation related zinc finger), a protein related to oxidative stress tolerance [44] and STZ (salt tolerance zinc finger), a TF implied in the response to salt stress [45].

The most repressed TF gene was ortholog to AtRL-5, whose function in Arabidopsis is unknown as mentioned previously (Table 1 and relative discussion). Two different members of the lateral organ boundary domain (LBD) family were repressed under drought conditions and corresponded to Arabidopsis AtLBD37 and AtLBD38. These two TFs are involved in the repression of anthocyanin biosynthesis and are important components in plant NO_3_^−^/N signaling [46]. The other down-regulated TFs also did not have any known role in response to drought. Interestingly, two highly repressed genes were orthologs to Arabidopsis ones involved in DNA repair: AtPCNA2 (proliferating cell nuclear antigen 2) [47] and AtBARD1 (breast cancer associated ring 1), that is also involved in stem cells development in the shoot apical meristem [48]. Other repressed TFs included orthologs to a basic helix-loop-helix (bHLH) TF with unknown function (probeset Ljwgs_140411.1), a cryptochrome interacting basic helix-loop-helix (CIB) transcription factor, a basic leucine zipper (AtBZIP10) and the ethylene response factor ERF72. The analysis presented indicates that while several highly induced genes encoding for TFs are part of the known response to abiotic stress, the down-regulated ones seem involved in several cellular processes apparently not related to stress. On the other hand, Table 2 also shows that the majority of the TF genes modulated in the WT plants under water deprivation were also modulated in the plastidic GS mutant *Ljgln2-2*. This indicates that the modulation of TF genes transcription in *L. japonicus* in response to drought is not dependent from the presence of plastidic GS. This is of particular interest since the *Ljgln2-2* mutant showed a peculiar response to recovery after drought, and an about three times higher number of genes were regulated in response to drought in this genotype compared to the WT [10]. It is also worth noticing that the mutant presented a higher extent of modulation of all the TF genes considered that were significantly regulated in both genotypes (Table 2). This probably reflects the higher level of stress that is received by the mutant at the same level of hydric deficit than the WT [10].

**Table 2:** Highly drought-modulated genes encoding for transcription factors. The fold-change (FC) is expressed as the log_2_ of the difference in relative expression levels between drought stress conditions and normal watering. Transcriptional data for the plastidic GS mutant *Ljgln2-2* are from Díaz *et al.* [10]. The WT and mutant plants used for this analysis showed similar levels of water loss (relative water content of 60.0% ± 2.5%). n.s.: not significant.

Probeset	log_2_ FC		Arabidopsis ortholog	Locus
	WT	*Ljgln2-2*		
Up-Regulated				
chr1.cm0104.32	3.70	5.37	NAC47	At3g04070
Ljwgs_036303.1	3.26	5.68	NAC domain RD26 TF	At4g27410
chr5.cm0052.19	3.01	5.95	ABR1, AP2 domain TF	At5g64750
chr5.cm0040.40.1	2.71	4.10	WRKY40	At1g80840
chr1.cm0023.10	2.66	n.s.	MYB96	At5g62470
chr3.cm0724.4	2.46	3.22	CCAAT box AtHAP2C	At1g72830
Ljwgs_031732.1	2.42	4.19	bZIP-1	At1g77920
Ljwgs_134387.1	2.16	n.s.	RAP2.10	At4g36900
chr4.cm0087.91	2.09	3.31	NAC100	At5g61430
chr4.cm0004.18	2.04	n.s.	AtOZF1	At2g19810
chr5.TM1598.10	2.02	n.s.	bHLH family TF	At1g09250
chr5.cm0200.109	2.01	3.91	STZ, ZAT10	At1g27730
chr3.cm0279.50	2.00	n.s.	AtNAC1	At1g56010
Down-regulated				
chr2.cm0249.113	−5.14	−7.27	AtRL-5	At1g19510
Ljwgs_140411.1	−2.78	−3.34	bHLH family TF	At1g72210
chr4.cm0128.29	−2.76	−4.05	AtLBD37	At5g67420
Ljwgs_049909.2	−2.59	−3.44	AtLBD38	At3g49940
Ljwgs_020020.1	−2.57	n.s.	AtPCNA2	At2g29570
chr1.cm0178.64	−2.27	−3.57	CIB1	At4g34530
chr6.cm0082.29	−2.19	n.s.	AtBARD1	At1g04020
Ljwgs_141699.1	−2.01	−2.36	ArERF72	At3g16770
Ljwgs_032996.1	−2.00	−3.59	AtBZIP10	At4g02640

## 3. Experimental

### 3.1. Plant Growth and Drought Treatments

*L. japonicus* (Regel) Larsen cv, Gifu seeds were initially obtained from Prof. Jens Stougaard (Aarhus University, Denmark) and then self-propagated at the University of Seville. The seeds were scarified and surface-sterilized, germinated in 1% agar Petri dishes, and transferred to pots using a 1:1 (v/v) mixture of vermiculite and sand as solid support. Five seedlings were planted in each pot and grown in a growth chamber under 16/8 h day/night, 20/18 °C, with a photosynthetic photon flux density of 250 μmol/m^2^·s and a constant humidity of 70%. Non-nodulated plants were watered with Hornum nutrient solution, containing 5 mM NH_4_NO_3 _and 3 mM KNO_3_ [8]. Drought conditions were applied by withholding irrigation to 35 days old plants. At this stage the plants had an average of seven fully expanded trifoils. The relative water content (RWC) of the leaves and the soil was measured each day. After 4 days of water deprivation the leaves were harvested, flash-frozen in liquid nitrogen and stored at −80 °C until use. The average RWC of the plants after four days of water deprivation was of 60.0% ± 2.5%, while the vermiculite/sand support used as soil showed a RWC of 31% ± 3.0%.

### 3.2. Measurement of Leaf and Soil Water Content

The water status of the leaves was expressed as the relative water content (RWC), calculated from the fresh weight (FW), dry weight (DW) and turgid weight (TW) of detached trefoils as follows: RWC (%) = 100 × (FW − DW)/(TW − DW). Dry weight was measured after incubation of the tissue overnight at 80 °C. Turgid weight was obtained after incubation of the detached trefoil for 8 h in water in a closed Petri dish. 

Soil RWC was defined as: RWC (%) = 100 × (FW − DW)/(SW − DW) where FW, DW and SW refer to soil fresh weight, oven-dry weight and weight at field capacity respectively.

### 3.3. RNA Extraction, Genechip Hybridization and qRT-PCR

Leaf material was immediately frozen in liquid nitrogen after harvest, homogenized with mortar and pestle and kept at –80 °C until use. Three independent biological replicates were used for qRT-PCR and transcriptomic analysis. A biological replicate consisted of a pool of tissue from five plants that were grown in the same pot. Total RNA was isolated using the hot borate method [9]. The integrity and concentration of the RNA preparations was checked using an Experion bioanalyzer (Bio-Rad) with RNA StdSens chips and a Nano-Drop ND-1000 (Nano-Drop Technologies) respectively. RNA samples were labeled using the One-Cycle Target labeling Kit (Affymetrix), hybridized to the Affymetrix Genechip® Lotus1a520343 and scanned according to the manufacturer’s instruction. MIAME compliant data were deposited at Array Express [49] as E-MEXP-3710. qRT-PCR validation of the microarray data using genes for proline metabolism was carried out as previously described [10]. 

## 4. Conclusions

In this paper we have shown that water deprivation induced an extensive reprogramming of the transcriptome in *L. japonicus*. This included several cellular processes such as the production of protective molecules, oxidative stress response, transport, cell wall restructuration, transcription and hormone metabolism, among others. The metabolic pathways that were significantly more regulated under drought conditions were related to carbon and amino acid metabolism. The transcriptional modulation of several genes involved in the control of the cell cycle was probably aimed to stop cell division and plant growth. Several highly drought responsive transcription factors were identified. Some of these genes were orthologs to Arabidopsis ones, with an important role in the response to abiotic stress, while others were probably peculiar to the Lotus drought stress response. Further experiments should be designed with the aim of characterizing these novel genes and the assessment of their eventual contribution to drought tolerance. The data set presented here also contributes to the global characterization of gene regulation in Lotus, a topic of great interest, recently approached for other purposes [50,51].

## Supplementary Files

Supplementary File 1 Supplementary File (XLS, 4966 KB)
